# Incidence of intoxication events and patient outcomes in Taiwan: A nationwide population-based observational study

**DOI:** 10.1371/journal.pone.0244438

**Published:** 2020-12-23

**Authors:** Chun-Kuei Chen, Yi-Ling Chan, Tse-Hsuan Su

**Affiliations:** 1 Department of Emergency Medicine, Chang Gung Memorial Hospital, Taoyuan, Taiwan; 2 College of Medicine, Chang Gung University, Taoyuan, Taiwan; 3 Department of Emergency Medicine, Chang Gung Memorial Hospital, Taoyuan, Taiwan; Technion - Israel Institute of Technology, ISRAEL

## Abstract

**Background:**

Intoxicated patients were frequently managed in the emergency departments (ED) with few studies at national level. The study aimed to reveal the incidence, outcomes of intoxications and trend in Taiwan.

**Methods:**

Adults admitted to an ED due to an intoxication event between 2006 and 2013 were identified using the Taiwan National Health Insurance Research Database. The rate of intoxication and severe intoxication events, mortality rate, hospital length of stay (LOS), and daily medical costs of these patients were analyzed. Changes over time were analyzed using Joinpoint models. Multivariable generalized regressions with GEE were used to assess the effect of sex, age, and presence of prior psychiatric illness.

**Results:**

A total of 20,371 ED admissions due to intoxication events were identified during the study period, and the incidence decreased with annual percentage change of 4.7% from 2006 to 2013. The mortality rate, hospital LOS, and daily medical costs were not decreased over time. Males and geriatric patients had more severe intoxication events, greater mortality rates, and greater daily medical costs. Patients with psychiatric illnesses had higher mortality rates and a longer hospital LOS, but lower daily medical expenses.

**Conclusion:**

From 2006 to 2013, there was a decline in the incidence of ED admission for intoxication events in Taiwan. Males, geriatric patients, and those with psychiatric illnesses had greater risks for severe intoxication and mortality.

## Introduction

Hospitalization due to intoxication is a burden to society and healthcare systems, although relatively little is known about the incidence of these events or patients’ outcomes at the nationwide level. The Taiwan poison control center’s previous study reported the incidence was 16 to 22 per 100,000 population, and the mortality rate was 5.7% from 1985 to 1996 [1]. Another mortality-survey-based study in Taiwan revealed the mortality rate of poisoning was 8.21 per 100,000 population [2]. A national study in Spain reported that intoxication events accounted for 0.66% of emergency department (ED) visits and had a mortality rate of 0.24% [3]. A study in Iceland reported these numbers as 0.39% and 0.09%, respectively [4].

The previous study in Taiwan only revealed the incidence and mortality rate of intoxication among the general populations. The incidence of intoxication among ED visits still lacks except for the studies from Spain and Iceland. Additionally, intoxication events differ from area to area. Our purpose was to determine the incidence, clinical outcomes, hospital length of stay, and medical cost of intoxicated patients admitted to EDs in Taiwan using data from the Taiwan National Health Insurance Database (NHIRD).

## Methods

### Data sources and study population

We conducted a retrospective cohort study using the Taiwan NHIRD. The Taiwan National Health Insurance (NHI) system is a single-payer, compulsory healthcare program covering more than 99.9% of Taiwan’s residents. This de-identified database contained registration files and original claim data, constructed in many data subset for research purposes [5]. Many published studies used this database [6–12]. A representative subset of two million people was randomly sampled from the 24 million beneficiaries of the Taiwan NHI between 2006 and 2013. There were no significant differences between this subset and all beneficiaries of the NHIRD. Data in the NHIRD are de-identified and contain complete individual clinical diagnoses, billed procedures, and prescriptions. This study was approved by the Chang Gung Medical Foundation Institutional Review Board, which waived the need for informed consent (IRB: 201900871B1).

### Study population, variables and definitions

All patients were eligible if they were aged 20 years and older and were admitted to an ED, with or without subsequent hospitalization, between 2006 and 2013. Patients admitted due to intoxication were identified using the diagnostic codes from the International Classification of Disease, Ninth Revision, Clinical Modification (ICD-9-CM; mainly including 960–989, E850-E869, E962, E972, E980-E982) or ever received antidotes. Because it’s difficult to differentiate whether the outcomes were more related to intoxicated or traumatic events, we excluded patients who presented to hospitals with trauma-related diagnoses. We have provided the demographic and clinical outcomes of these trauma-related intoxicated patients in the supplemental tables (S1 and S2 Tables). To assure adequate long-term follow-up of all patients, those admitted after September 30, 2013 were also excluded. An event of severe intoxication was defined by admission to an intensive care unit (ICU), or receipt of an inotropic agent, cardiopulmonary resuscitation (CPR), or mechanical ventilation during hospitalization.

All data from inpatient and outpatient databases were from 1 year before the index medical visit. Age, sex, socioeconomic status, and associated comorbidities (hypertension, diabetes mellitus, heart failure, ischemic heart diseases, obstructive lung diseases, cerebrovascular disease, liver diseases, chronic renal disease, psychiatric illness, and malignancy), and previous intoxicated events were recorded. All comorbidities were recorded if the associated ICD-9-CM diagnostic codes were present during at least one hospitalization or two outpatient visits from 1 year prior to the index ED visit. Events that occurred during hospitalization, including respiratory failure, shock, CPR, and receipt of hemodialysis or hemoperfusion, were recorded. Respiratory failure was defined as the need for mechanical ventilatory support, and shock as the need for an inotropic agent.

### Outcomes

The primary outcomes include the incidence of intoxicated events, the incidence of severely intoxicated events, and in-hospital mortality rate. The incidence of intoxication events was determined as the number of ED admissions for intoxication events divided by the total number of ED admissions. The secondary outcomes were hospital length of stay (LOS) and daily medical costs during hospitalization (calculated as total medical costs divided by total LOS).

### Statistical analysis

Continuous variables are presented as median ± interquartile range (IQR), and categorical variables presented with percentages. Continuous data were compared using the Wilcoxon signed-rank test, and categorical data were compared using the X^2^ tests for significance. The annual incidence of intoxication events, severe intoxication events, and in-hospital mortality rate were calculated. Using data from the 2006 patient cohort as the reference, the annual percentage change over time was calculated using Joinpoint regression from the Joinpoint Regression Program Version 4.8.0.1 (Statistical Methodology and Applications Branch, Surveillance Research Program, National Cancer Institute, USA). Temporal trends were assessed using a multivariable generalized regression model based on a generalized estimating equation (GEE) that accounted for hospital clustering and adjusted for baseline characteristics at the hospital level.

We are interested in the incidence and outcomes based on different sex, age group (young: 20–39 years; middle-age: 40–65 years; senior: >65 years), and prior history of psychiatric illness. We used multivariable generalized regression models with GEE to calculate the risk ratios, with or without variables adjustment, based on sex, age group, and prior history of psychiatric illness. The GEE was used with a Poisson distribution and the log link for analysis of LOS, and with the Gamma distribution and the log link for analysis of daily medical costs. As to the mortality outcome, we used binary distribution and the logit link for analysis. Independent working correlation matrix were used in the model. The models were adjusted with the year of visits, age, sex, socioeconomic status, comorbidities (diabetes, hypertension, history of ischemic heart disease or heart failure, malignancy history, obstructive lung disease, cerebrovascular diseases, chronic kidney disease, liver diseases, psychiatric illness), and previous intoxicated events in the prior year. Bonferroni correction was used to adjust for multiple comparisons between different age groups, and the results were reported as corrected 95% confidence intervals (CIs). Statistical analyses were performed using SAS version 9.4 and a 2-tailed *p-*value less than 0.05 was considered significant.

## Results

### Baseline characteristics

**Fig 1:**
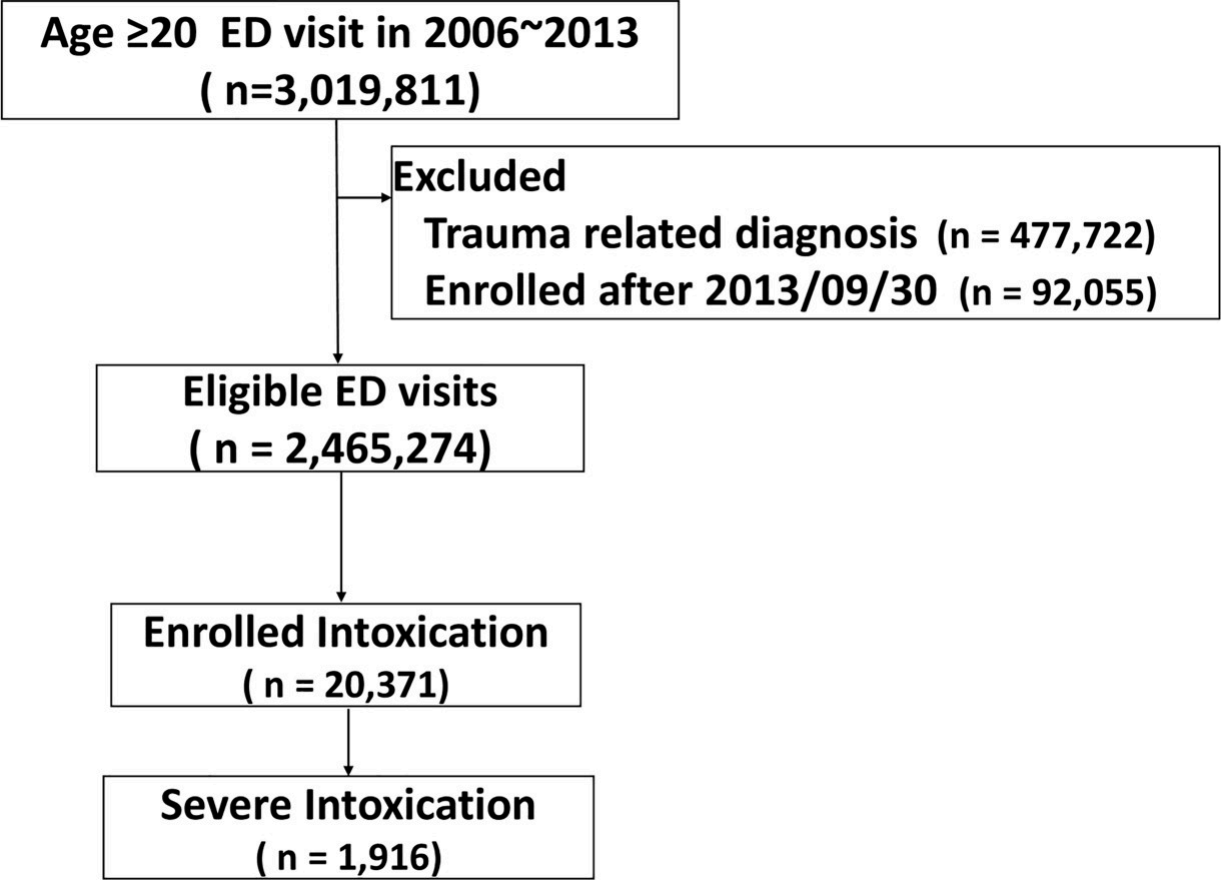
Flowchart of study cohort.

We identified 20,371 ED admissions due to intoxication events or severe intoxication events among 2,465,274 total ED visits between 2006 and 2013 (Fig 1). There were 18,455 admissions (90.6%) due to intoxication and 1916 (9.41%) admissions due to severe intoxication. The median age of intoxicated and severely intoxicated patients were 44 (IQR, 33–58) and 53 years (IQR, 38–72), respectively (*p* < .0001). There were more females admitted for intoxication (n = 8962, 56%), but more males admitted for severe intoxication (n = 1041, 54%). A total of 10.7% of the intoxication events and 36% of the severe intoxication events led to inter-hospital transfer. Patients who experienced severe intoxication had more comorbidities (Table 1).

**Table 1:** Demographic data of the study cohort.

Characteristics	Overall, intoxication n = 20,371	Severe intoxication n = 1,916	Non-severe intoxication n = 18,455	*p value*
**Patient Characteristics**				
Age, years–median (IQR)	44 (33–58)	53 (38–72)	43 (32–57)	< .0001
Male (%)	44.0	54.3	42.9	< .0001
**Hospital Levels**				0.2690
Medical centers	26.1	24.5	26.2	
Regional hospitals	46.2	52.0	45.6	
Local hospitals	27.7	23.5	28.1	
Inter-hospital transfer	10.7	36.0	8	< .0001
**Socioeconomic status: monthly income (US Dollars), %**				. < .0001
Dependent	20.8	23.9	20.5	
< 667	31.6	35.9	31.2	
667–1334	37	36.1	37.1	
> 1334	10.6	4.2	11.2	
**Medical history (%)**				
Hypertension	18.0	29.3	16.9	< .0001
Diabetes mellitus	9.9	17.6	9.1	< .0001
Ischemic heart disease/heart failure	10.8	21.5	9.7	< .0001
Malignancy	2.8	4.7	2.6	< .0001
Obstructive lung disease	3.9	7.9	3.5	< .0001
Cerebrovascular disease	0.4	0.9	0.4	0.0212
Liver disease	7.4	10.3	7.1	< .0001
Chronic renal disease	1.7	4.3	1.4	< .0001
Psychiatric illness	34.2	40.5	33.6	< .0001
Prior intoxication†	4.6	6.2	4.4	0.0026

† defined as previous relative ICD9 or antidote use in 1 year.

### In-hospital treatment and clinical outcomes

More than 80% of the patients with severe intoxication were admitted to an ICU, and stay in the ICU for 3 days (IQR 2–6). Approximately half of these patients with severe intoxication developed respiratory failure, 42.3% with shock, and 11.4% with cardiac arrest that required CPR. Only 10.9% of patients received hemodialysis or hemoperfusion. 218 patients received CPR. Among them, 83 patients were not admitted to ICU because most of them (N = 76, 91.6%) died before ICU admission. Besides, 956 patients received mechanical ventilation, but 175 patients were not admitted to ICU. More than half of them (N = 98, 56.0%) died before ICU admission. On the contrary, three-quarters of the patients with intoxication were directly discharged from the ED, and this group also had a much shorter hospital LOS. Less than one percent of the patients died in the ED before hospitalization. The daily medical costs were significantly lower for patients with intoxication than severe intoxication (Table 2).

**Table 2:** In-hospital treatment and outcomes in intoxicated patients.

	Overall, intoxication n = 20,371	Severe intoxication n = 1,916	*p value*
**In-hospital treatments (%)**			
Cardiopulmonary resuscitation	1.1	11.4	< .0001
Mechanical ventilator support	4.7	49.9	< .0001
Defibrillation	0.3	3.2	< .0001
Inotropic agents	4.0	42.3	< .0001
Renal replacement therapy	1.4	10.9	< .0001
ICU admission	7.7	81.8†	< .0001
Hospitalization	23.5	83.4†	< .0001
**Clinical outcomes**			
Mortality (%)	2.6	21.6	< .0001
Total medical costs (USD‡), median (IQR)	96 (45–290)	1801 (825–4099)	< .0001
Hospital length of stay, days, median (IQR)	1 (1–2)	7 (3–15)	< .0001
Daily Medical cost (USD), median (IQR)	76 (41–125)	241 (169–345)	< .0001

†:18.2% (n = 349) of patients with severe intoxication didn’t admitted to the ICU admission. Among them, 36.4% (n = 127) ever admitted to other hospital units.

‡1 United States Dollar (USD) = 30 New Taiwan Dolloars.

### Trend of incidence in intoxication, severe intoxication, and mortality

**Fig 2:**
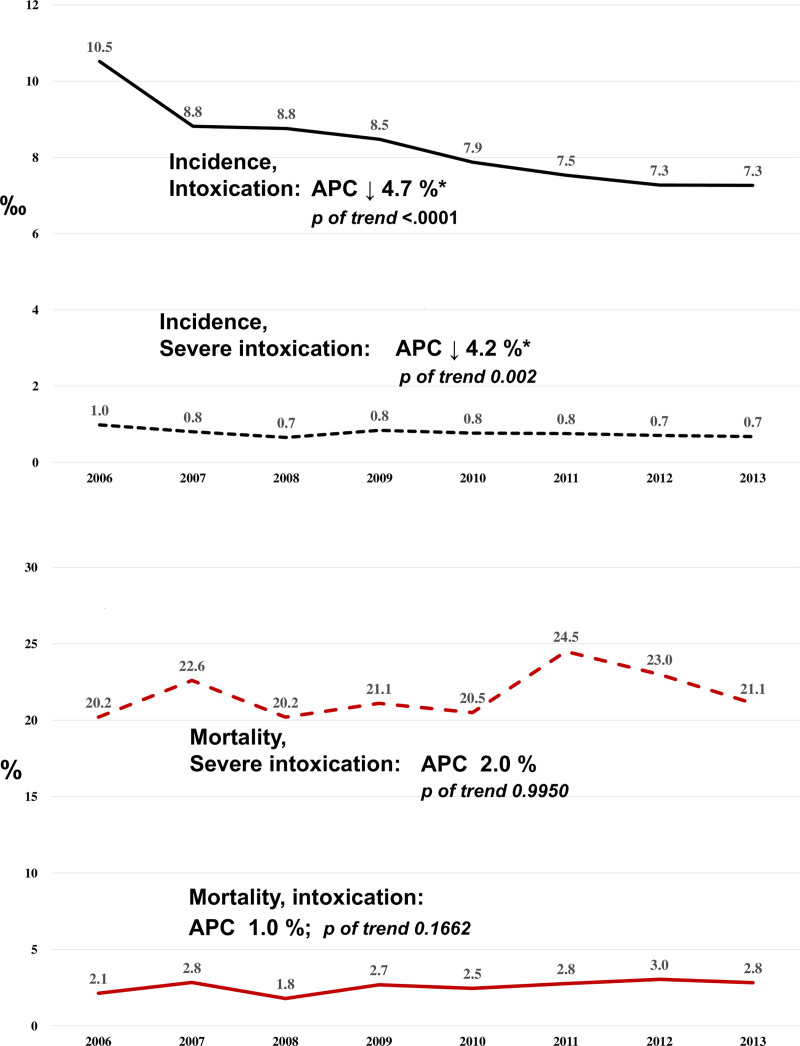
**A.** Trend of Incidence in intoxicated and severe intoxicated patients. Both the incidence of intoxicated events, and the incidence of severe intoxicated events were decreasing over times. The APC were decreased 4.7% in the incidence of intoxicated events, and 4.2% in the severe intoxicated. Abbreviations: APC: annual percentage change; **B.** Trend of the mortality in intoxicated and severe intoxicated patients. There was no significant trend change of mortality in both the intoxicated or severe intoxicated events.

The overall incidence of intoxication was 8.3 per 1000 of ED visits and 0.8 per 1000 for severe intoxication. The incidence of intoxication decreased from 10.5 per 1000 in 2006 to 7.3 per 1000 in 2013 (*p* for trend < 0.0001). The annual percent change decreased by 4.7 percent per year (p < .001, Fig 2A). The incidence of severe intoxication decreased from 1.0 per 1000 to 0.7 per 1000 from 2006 to 2013 (*p* for trend = 0.002, Fig 2A), corresponding to an annual percent change decreasing of 4.1% per year (p = 0.039). The mortality rate of intoxication and severe intoxication remained unchanged during the study period (Fig 2B).

#### Trend of hospital LOS, and daily medical cost

**Fig 3:**
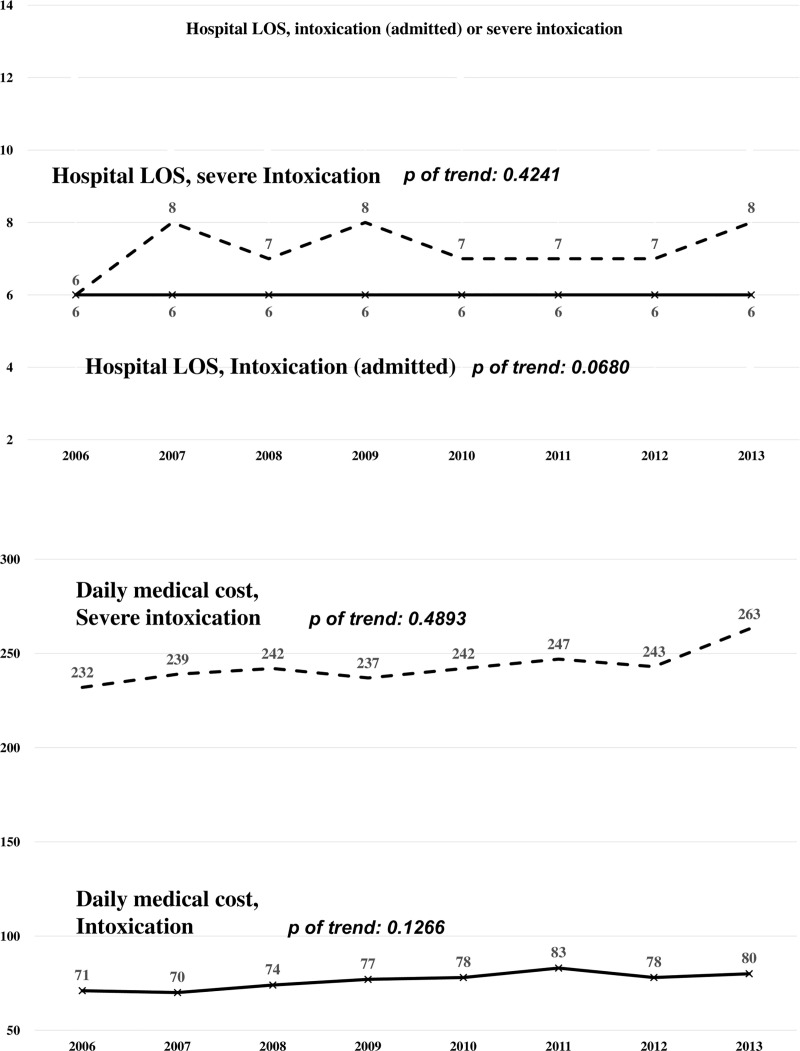
**A.** Trend of the Hospital Length of Stay (LOS) in patients with intoxication and severe intoxication. The LOS was 6 days in intoxicated patients and 7 days in patients with severe intoxication. There was no trend changed in the LOS in patients with intoxicated and severe intoxicated. *The LOS of intoxicated were calculated from patients ever admitted to hospitals. **B.** Trend of the daily medical costs in patients with intoxication and severe intoxication. The trend of the daily medical costs in patients with intoxication and severe intoxication were similar between 2006 and 2013.

For patients hospitalized (n = 4785, 23.5%), the median hospital LOS was six days (IQR: 4–12), and there was no significant change in the LOS over time (*p* for trend = 0.068, Fig 3A). For patients with severe intoxication, the hospital LOS was seven days (IQR: 3–15) without significant change over time (*p* for trend = 0.4241, Fig 3A). Daily medical costs remained unchanged over time for both groups (Fig 3B).

### Risk ratio on different sex, age group, and previous psychiatric illness

Multivariable generalized regression with GEE based on sex (Table 3) showed that females had a higher incidence of intoxication events (adjusted risk ratio (aRR) [95%CI]: 0.91 [0.87–0.95]), but males had a higher incidence of severe intoxication events (aRR: 1.32 [1.19–1.46]). The incidence of intoxication events in both sexes decreased over time (annual percentage change: males: 3.6%; females: 5.5%; *p* of interaction, 0.0568, S1 Fig). The overall mortality was higher in males (aRR: 1.58 [1.32–1.89]). For those with severe intoxication events, males had marginally higher mortality (crude risk ratio [cRR]: 1.24 [1.00–1.54], *p* = 0.0474), but this risk was similar after adjustment for comorbidities and confounders (aRR: 1.15 [0.92–1.43]). Sex was unrelated to hospital LOS (aRR: 1.02 [0.80–1.32]), but males had higher daily medical costs (aRR: 1.13 [1.08–1.19]).

**Table 3:** Risk ratio based on sex.

Male versus Female (Ref group: Female)	Crude Risk ratios RR (95% CI)	Adjusted Risk ratios ‡ RR (95% CI)
Incidence of the intoxication	0.83 (0.79, 0.87)****	0.91 (0.87, 0.95)****
Incidence of the severe intoxication	1.26 (1.14, 1.39)***	1.32 (1.19, 1.46)****
Mortality, overall	1.74 (1.47, 2.06)****	1.58 (1.32, 1.89)****
Mortality of the severe intoxication	1.24 (1.00, 1.54)*	1.15 (0.92, 1.43)
Hospital length of stay†	0.99 (0.75, 1.31)	1.02 (0.80, 1.32)
Daily medical cost †	(1.09, 1.21) ****	(1.08, 1.19)****

† Only analyzed patient with severe intoxicated or admitted to the hospitals.

‡. Adjusted model were adjusted with year of hospital visit, age, socioeconomic status, comorbidities (diabetes, hypertension, history of ischemic heart disease or heart failure, cerebrovascular disease, obstructive lung diseases, chronic kidney disease, liver disease, psychiatric illness, history of malignancy, and previous intoxicated events in the prior year.

*p < 0.05

** p<0.01

*** p<0.001

**** p< 0.0001.

Multivariable generalized regression with GEE based on age (Table 4) indicated that relative to the young age group, the risk of an intoxication event was slightly higher in the middle-age group (aRR: 1.06 [1.01–1.12], *p* = 0.0058), but lower in the senior age group (aRR: 0.68 [0.63–0.74], *p* < 0.0001). On the other hand, compared with the young age group, the senior age group had a higher risk of a severe intoxication. The senior age group also had the greatest daily medical costs, and greater overall mortality compared with the young age group (aRR: 4.03 [2.88–5.64]) and the middle-age group (aRR: 1.73 [1.18–2.52]). The three age groups had similar hospital LOS.

**Table 4:** Risk ratio based on different age groups.

	Crude Risk Ratios RR (95%CI)	Adjusted Risk Ratios‡ RR (95%CI)
**40–65 versus 20–39 (Ref group: 20–39)**		
Incidence of the intoxication	0.99 (0.95, 1.04)	1.06 (1.01, 1.12)**
Incidence of the severe intoxication	1.30 (1.13, 1.50)****	1.27 (1.08, 1.48)**
Mortality, overall	1.51 (1.11, 2.07)**	1.50 (1.09, 2.06)**
Mortality of the severe intoxication	1.43 (0.96, 2.14)	1.43 (0.96, 2.13)
Hospital length of stay†	1.36 (0.92, 2.00)	1.44 (0.95, 2.18)
Daily medical cost †	1.09 (0.99, 1.19)	1.10 (1.01, 1.21)*
**> 65 versus 40–65 (Ref group: 40–65)**		
Incidence of the intoxication	0.55 (0.51, 0.59)****	0.64 (0.59, 0.68)****
Incidence of the severe intoxication	1.22 (1.08, 1.39)***	1.09 (0.95, 1.25)
Mortality, overall	3.29 (2.54, 4.26)****	2.70 (2.00, 3.63)****
Mortality of the severe intoxication	1.49 (1.09, 2.05)**	1.73 (1.18, 2.52)**
Hospital length of stay†	0.76 (0.51, 1.13)	0.77 (0.55, 1.09)
Daily medical cost †	1.07 (0.99, 1.16)	1.10 (1.02, 1.19)**
**>65 versus 20–39 (Ref group: 20–39)**		
Incidence of the intoxication	0.54 (0.51, 0.59)****	0.68 (0.63, 0.74)****
Incidence of the severe intoxication	1.59 (1.38, 1.84)****	1.38 (1.15, 1.64)****
Mortality, overall	4.97 (3.72, 6.65)****	4.03 (2.88, 5.64)****
Mortality of the severe intoxication	2.14 (1.47, 3.12)****	2.46 (1.63, 3.73)****
Hospital length of stay†	1.03 (0.71, 1.50)	1.10 (0.79, 1.55)
Daily medical cost †	1.17 (1.06, 1.28)***	1.22 (1.11, 1.34)****

† Only analyzed patient with severe intoxicated or admitted to the hospitals.

‡ Adjusted model were adjusted with year of hospital visit, sex, socioeconomic status, history of diabetes, hypertension, history of ischemic heart disease or heart failure, cerebrovascular disease, obstructive lung diseases, chronic kidney disease, liver disease, psychiatric illness, history of malignancy, and previous intoxicated events in the prior year.

§ Bonforroni method was used to correct for multiple comparison between different age group. We reported adjusted CI after Bonforroni correction.

*p < 0.05

** p<0.01

*** p<0.001

**** p< 0.0001.

Multivariable generalized regression with GEE also indicated that history of a psychiatric illness increased the risk of an intoxication event (aRR: 2.72 [2.56–2.89], *p* < 0.0001, Table 5) and a severe intoxication event (aRR: 2.73 [2.42–3.08], *p* < 0.0001). These patients also had greater overall mortality (aRR: 1.30 [1.08–1.56], *p* = 0.0057) and longer hospital LOS (aRR: 1.40 [1.07–1.85]; *p* = 0.0155), but lower daily medical costs (aRR: 0.93 [0.89–0.98]). The mortality rate from a severe intoxication event was similar in those with and without history of a psychiatric illness.

**Table 5:** Risk ratio based on history of psychiatric illness.

With versus without psychiatric illness (Ref group: without diseases)	Crude Risk ratios RR (95% CI)	Adjusted Risk ratios ‡ RR (95% CI)
Incidence of the intoxication	2.33 (2.20, 2.47)****	2.72 (2.56, 2.89)****
Incidence of the severe intoxication	2.99 (2.68, 3.33)****	2.73 (2.42, 3.08)****
Mortality, overall	1.29 (1.09, 1.53)**	1.30 (1.08, 1.56)**
Mortality of the severe intoxication	0.89 (0.72, 1.10)	0.95 (0.75, 1.19)
Hospital length of stay†	1.38 (1.04, 1.83)*	1.40 (1.07, 1.85)*
Daily medical cost †	0.89 (0.84, 0.94)****	0.93 (0.89, 0.98)**

† Only analyzed patient with severe intoxicated or admitted to the hospitals.

‡. Adjusted model were adjusted with year of hospital visit, sex, age, socioeconomic status, comorbidities (diabetes, hypertension, history of ischemic heart disease or heart failure, cerebrovascular disease, obstructive lung diseases, chronic kidney disease, liver disease, history of malignancy, and previous intoxicated events in the prior year.

*p < 0.05

** p<0.01

*** p<0.001

**** p< 0.0001.

## Discussion

In this nationwide population-based study, we demonstrated that the incidence of intoxicated or severe intoxicated patients in Taiwan declines significantly between 2006 and 2013 (annual percentage change decreased by 4.7% & 4.2%). However, the mortality, hospital LOS, and daily medical costs related to intoxication events remained similar during this period. The causes of decline could be multifactorial, including medical and health policy, social-economic status, and suicide prevention work. The suicide prevention center of Taiwan was set up in 2005, and there were two phases of the suicide prevention program conducted from 2005 to 2013. These programs decreased the suicide rate in Taiwan [13]. The suicide prevention hotline may also play roles in reducing emotional distress and suicide risk [14]. It was set up at the end of 2005 and has been undertaken by the Taipei Lifeline association since 2009. Besides, the Gross domestic product (GDP) was also increasing during the study period (2006 to 2013). All these possible causes might explain part reasons for the declined incidence, but the causal relationship could not be made from our paper and require further study. Another significant finding was that the risks for mortality from intoxication were higher in males (58% increased risk), the elderly (303%), and those with a previous psychiatric illness (30%).

We found that intoxication events accounted for 8.8 per 1000 ED admissions, similar to previous studies. In particular, intoxication events accounted for about 1 to 7 per 1000 ED admissions in Spain and Oslo [3,15]. Although Taiwan had a slightly greater incidence, its annual percentage change decreased by about 4% between 2006 and 2013. We found that most patients in Taiwan who had intoxication events were directly discharged from the ED, similar to other studies (Taiwan: 76%; National Poison Data System [NPDS]: 47–51%; other studies: 55–79%) [3,4,15–23]. These patients presumably had minor intoxication events [24], received antidotes promptly, rapidly eliminated the toxins, or absorbed smaller amounts of toxins.

In contrast, it is not similar for patients severely intoxicated. In particular, 36% of patients with severe intoxication were transferred to different hospitals, about 3.5-times more often than patients who had intoxication. The possible reasons for this include the lack of toxicologists, the need for specific laboratory tests or antidotes, or the presence of a serious condition that required intensive care. Moreover, 81.8% (N = 1567) of our severely intoxicated patients were admitted to an ICU. Among severely intoxicated patients who were not admitted to an ICU, only 36.4% were ever admitted to wards. It may be due to a lack of ICU beds, because the patient stabilized or expired quickly while in the ED, or because the patient signed a do-not-resuscitate order or refused intensive care.

Our overall in-hospital mortality rate was much higher than reported in other countries (Taiwan: 2.6%, other countries: 0.1–1.3%), as was our mortality rate from severe intoxication (Taiwan: 21.6%, other countries: 2–9%) [3,4,25,26]. The most likely reason is the higher proportion of severe intoxication events or greater severity of intoxication in our population [16–24,27–29]. Our mean ICU stay (3 days) and hospital LOS (7 days) was longer than reported in studies from The Netherlands and Hong Kong (ICU: 0–1.3 days; hospital LOS: 1–3 days) [25,29,30]. If we calculate the ratio between the number of patients receiving different in-hospital treatments to patients receiving mechanical ventilation, the ratio of the patients receiving inotropic agents to patients receiving mechanical ventilation was 0.848:1. The ratios of the number of patients who received CPR and hemodialysis to the number who received intubation were 0.228:1 and 0.309:1, respectively. Analysis of data from the NPDS [16–23] for intoxicated patients older than 20 years between 2006 and 2013 indicated that the ratios of the numbers of patients who received an inotropic agent, CPR, and hemodialysis to the number of patients who received mechanical ventilation were 0.269:1, 0.045:1 and 0.125:1, respectively. Although we are unable to directly compare the severity of these intoxicated patients between different studies, the ratio between the number received specific in-hospital treatment (inotropic agents, CPR, hemodialysis) to the number received mechanical ventilation may indirectly revealed the severity of the study population. The need for more intensive interventions in our population may be because highly toxic pesticides, such as paraquat, were available in Taiwan during the study period. This may also account for the higher mortality in our population [12,31,32]. Because our population had longer ICU stays, higher percentages of patients who received aggressive medical or resuscitation treatments, and greater exposure to highly toxic pesticides, it is reasonable that our population also had a higher percentage of patients with severe intoxication.

Multivariable regression based on sex, age, and previous psychiatric illness indicated that females were 9% more likely to present with intoxication, but males were 32% more likely to present with severe intoxication. Previous studies from the United States, Iran, and Nordic countries reported similar patterns [16,33–36]. However, our examination of patients with severe intoxication indicated that sex was unrelated to survival. The incidence of severe intoxication among different age groups also varied in previous studies [28,33,37]. We found that elderly patients were more likely to die from severe intoxication. In particular, we demonstrated that the middle-age group had the highest incidence of severe intoxication, but the elderly group had the most increased mortality from severe intoxication. Previous studies showed that geriatric patients were intoxicated mostly by accident [38]. However, they were also more susceptible to intoxication because they tend to have more comorbidities [39].

We found that patients with previous psychiatric illnesses were more likely present to an ED with intoxication, have more severe intoxication, and die from intoxication. These results are consistent with previous studies that patients with psychiatric illness had higher drug overdose [40–43]. Additionally, in our study, these patients had longer hospital LOS, but lower daily medical expenses. We are uncertain about the reasons for their lower medical expenses. In fact, it seems likely that many of these patients required prolonged periods of psychiatric adjustment, evaluation, or admission to a psychiatric ward before resolution because they have an increased risk of a subsequent episode of self-harm or intoxication [44–46].

### Strengths and limitations

A major strength of this study is that it was a nationwide population-based study which examined the incidence and clinical outcomes of intoxication events in adults who ever visited the ED and the temporal trend change from 2006 to 2013. We also evaluated the difference in patients based on sex, age, and psychiatric illness. Nevertheless, our study had some limitations. First, we extracted data from an insurance claims database, which did not provide clinical information unrelated to its original purposes, such as the reasons for the intoxications, the details of substance ingestion, associated personal history, and laboratory results. Thus, we were unable to identify the names or amounts of different intoxicants nor the reasons for ingestion. The drug-drug interaction and the effect of polypharmacy could not be evaluated in this study as well. Regardless, our analysis from the insurance claims database was reliable and provided a nationwide perspective. Second, the medical history of psychiatric illness accounted for a significant number of patients included in this study, which was a risk factor for severe intoxication. The Taiwan National Health Isurance (NHI) system provided only ICD9-CM but did not provide more specific details concerning the different psychiatric pathologies. Therefore, we have limited information in evaluating whether intoxication is more common in certain psychiatric pathologies and less in others, requiring further validation study in the future. Third, our study excluded trauma-related diagnoses. Some patients might present to ED with trauma-related diagnoses but also were intoxicated. The numbers were a total of 2,205, around 0.46% of trauma cases. These exclusions might cause certain biases. Fourth, we did not have all relevant clinical data for the patients, such as body temperature, heart rate, blood pressure, and level of consciousness. Instead, we classified patients with severe intoxication based on the treatment received. Although we did not classify patients with transiently unstable vital signs as severe cases, we considered intoxicated patients who required an inotropic agent, mechanical respiratory support, CPR, or ICU admission as being truly severe. Fifth, because we identified intoxication events based on ICD9 codes and the antidotes administered, there may have been some misclassification. However, previous studies based on similar databases showed reliable results [2,12,47–51]. Our current research thus provides an overview of intoxication events in Taiwan between 2006 and 2013. We are planning the development of an intoxication registry at the national level with associated parameters for future studies.

## Conclusions

We found that the incidence of intoxication events in Taiwan decreased from 2006 to 2013. However, the mortality rate remained high and without changes over time during the study period. There were also only limited changes in hospital LOS and daily medical costs. Males, the elderly, and those with previous psychiatric illnesses had a greater risk of severe intoxication. Therefore, clinicians should be alert to the possibility of severe intoxication during evaluation and administer appropriate intensive care.

## Supporting information

S1 Fig Risk ratio on sex and incidence. (TIF)

S1 Table Demographic data of the study cohort. (DOCX)

S2 Table In-hospital treatment and outcomes in intoxicated patients. (DOCX)
