# Supplementary figures and images for: Incidence of intoxication events and patient outcomes in Taiwan: A nationwide population-based observational study
Source: PLoS One. 2020 Dec 23;15(12):e0244438. doi: 10.1371/journal.pone.0244438 (PMC7757892; doi:10.1371/journal.pone.0244438)

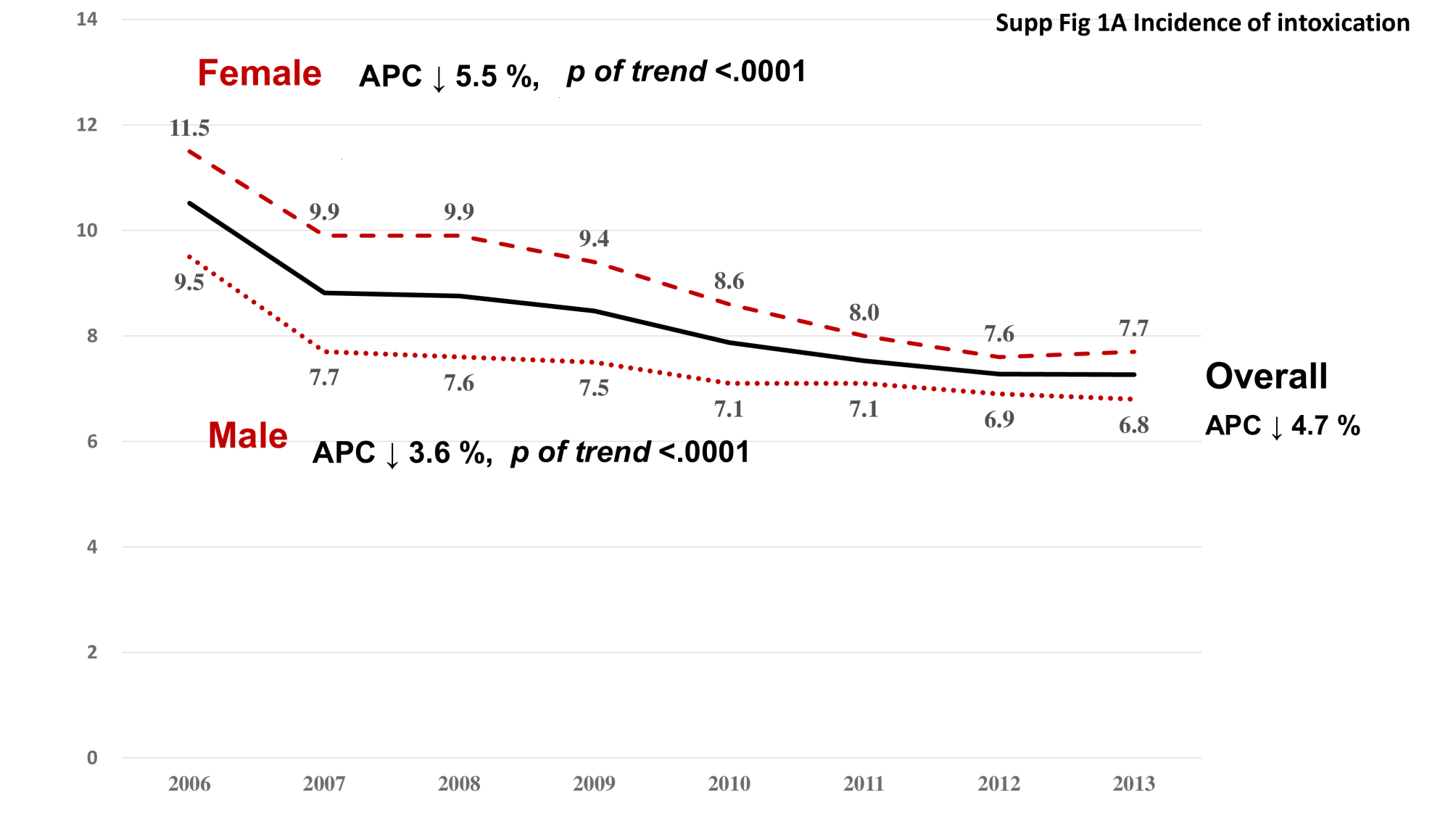

Supplement: S1 Fig — (TIF) [file pone.0244438.s001.tif]
