# Supplementary material for: Incidence of intoxication events and patient outcomes in Taiwan: A nationwide population-based observational study
Source: PLoS One. 2020 Dec 23;15(12):e0244438. doi: 10.1371/journal.pone.0244438 (PMC7757892; doi:10.1371/journal.pone.0244438)
Supplement: S1 Table — (DOCX) [file pone.0244438.s002.docx]

**Supplemental Tables 1: Demographic data of the study cohort**

|  | Both non-trauma/trauma | | Non-Traumatic | | Traumatic | |
| --- | --- | --- | --- | --- | --- | --- |
| Characteristics | Overall, intoxication  n = 22,576 | Severe  intoxication n = 2,064 | Overall, intoxication  n = 20,371 | Severe  intoxication n = 1,916 | Overall, intoxication  n = 2,205 | Severe  intoxication n = 148 |
| **Patient Characteristics** |  |  |  |  |  |  |
| Age, years – median (IQR) | 44 (32-58) | 53 (38-72) | 44 (33-58) | 53 (38-72) | 44 (31-58) | 51 (38-70) |
| Male (%) | 44.4 | 54.0 | 44.0 | 54.3 | 48.0 | 49.3 |
| **Hospital Levels** |  |  |  |  |  |  |
| Medical centers | 25.2 | 23.9 | 26.1 | 24.5 | 17.6 | 16.2 |
| Regional hospitals | 46.2 | 51.9 | 46.2 | 52.0 | 45.6 | 50.7 |
| Local hospitals | 28.6 | 24.2 | 27.7 | 23.5 | 36.9 | 33.1 |
| Inter-hospital transfer | 11 | 36.9 | 10.7 | 36.0 | 14.2 | 48.0 |
| **Socioeconomic status:**  **monthly income (US Dollars), %** |  |  |  |  |  |  |
| Dependent | 20.7 | 23.6 | 20.8 | 23.9 | 20.3 | 20.3 |
| < 667 | 31.4 | 36.2 | 31.6 | 35.9 | 29.8 | 41.2 |
| 667 – 1334 | 37.1 | 36 | 37 | 36.1 | 37.7 | 35.1 |
| > 1334 | 10.7 | 4.1 | 10.6 | 4.2 | 12.2 | 3.4 |
| **Medical history (%)** |  |  |  |  |  |  |
| Hypertension | 17.8 | 29.2 | 18.0 | 29.3 | 15.5 | 27.7 |
| Diabetes mellitus | 9.7 | 17.1 | 9.9 | 17.6 | 7.5 | 9.5 |
| Ischemic heart disease/heart failure | 10.5 | 21.3 | 10.8 | 21.5 | 8.1 | 18.2 |
| Malignancy | 2.8 | 4.7 | 2.8 | 4.7 | 2.6 | 3.4 |
| Liver disease | 7.4 | 10.3 | 7.4 | 10.3 | 6.8 | 10.1 |
| Chronic renal disease | 1.6 | 4.2 | 1.7 | 4.3 | 0.8 | 3.4 |
| Psychiatric illness | 33.4 | 40.6 | 34.2 | 40.5 | 25.7 | 41.9 |
| Prior intoxication | 4.6 | 6.0 | 4.6 | 6.2 | 4.5 | 3.4 |
