# Supplementary material for: Incidence of intoxication events and patient outcomes in Taiwan: A nationwide population-based observational study
Source: PLoS One. 2020 Dec 23;15(12):e0244438. doi: 10.1371/journal.pone.0244438 (PMC7757892; doi:10.1371/journal.pone.0244438)
Supplement: S2 Table — (DOCX) [file pone.0244438.s003.docx]

**Supplemental Tables 2: In-hospital treatment and outcomes in intoxicated patients**

|  | Both non-trauma/trauma | | Non-Traumatic | | Traumatic | |
| --- | --- | --- | --- | --- | --- | --- |
|  | Overall, intoxication  n = 22,576 | Severe  intoxication n = 2,064 | Overall, intoxication  n = 20,371 | Severe  intoxication n = 1,916 | Overall, intoxication  n = 2,205 | Severe  intoxication n = 148 |
| **In-hospital treatments (%)** |  |  |  |  |  |  |
| Cardiopulmonary resuscitation | 1 | 11.2 | 1.1 | 11.4 | 0.6 | 9.5 |
| Mechanical ventilator support | 4.6 | 49.8 | 4.7 | 49.9 | 3.3 | 48.6 |
| Defibrillation | 0.3 | 3.1 | 0.3 | 3.2 | 0.1 | 1.4 |
| Inotropic agents | 3.9 | 42.6 | 4.0 | 42.3 | 3.1 | 45.9 |
| Renal replacement therapy | 1.3 | 10.4 | 1.4 | 10.9 | 0.4 | 4.7 |
| ICU admission | 7.5 | 82 | 7.7 | 81.8 | 5.7 | 85.1 |
| Hospitalization | 21.4 | 89.8 | 23.5 | 83.4 | 20 | 95.3 |
| **Clinical outcomes** |  |  |  |  |  |  |
| Mortality (%) | 2.5 | 21.5 | 2.6 | 21.6 | 1.6 | 20.3 |
| Total medical costs (USD‡),  median (IQR) | 94  (42-293) | 1,829  (866-4,183) | 96  (45-290) | 1,801  (825-4,099) | 69  (32-337) | 2,482  (1,204-5,818) |
| Hospital length of stay,  days, median (IQR) | 1 (1-2) | 7 (3-16) | 1 (1-2) | 7 (3-15) | 1 (1-2) | 9 (4-25) |
| Daily Medical cost (USD),  median (IQR) | 75  (38-125) | 241  (169-347) | 76  (41-125) | 241  (169-345) | 55  (31-124) | 246  (160-382) |

‡1 United States Dollar (USD) = 30 New Taiwan Dolloars.
